# Fluorescence microscopy shadow imaging for neuroscience

**DOI:** 10.3389/fncel.2024.1330100

**Published:** 2024-02-15

**Authors:** V. V. G. Krishna Inavalli, Virginia Puente Muñoz, Jonathan E. Draffin, Jan Tønnesen

**Affiliations:** ^1^Center for Cancer Immunology, University of Southampton, Southampton, United Kingdom; ^2^Department of Neurosciences, Faculty of Medicine and Nursing, University of the Basque Country (UPV/EHU), Leioa, Spain; ^3^Neuronal Excitability Lab, Achucarro Basque Center for Neuroscience, Leioa, Spain; ^4^Aligning Science Across Parkinson’s (ASAP), Collaborative Research Network, Chevy Chase, MD, United States; ^5^Instituto Biofisika (CSIC/UPV), Leioa, Spain

**Keywords:** fluorescence microscopy, shadow imaging, brain extracellular space, super-resolution microscopy, neuroscience, STED microscopy, two-photon imaging, SUSHI

## Abstract

Fluorescence microscopy remains one of the single most widely applied experimental approaches in neuroscience and beyond and is continuously evolving to make it easier and more versatile. The success of the approach is based on synergistic developments in imaging technologies and fluorophore labeling strategies that have allowed it to greatly diversify and be used across preparations for addressing structure as well as function. Yet, while targeted labeling strategies are a key strength of fluorescence microscopy, they reciprocally impose general limitations on the possible types of experiments and analyses. One recent development that overcomes some of these limitations is fluorescence microscopy shadow imaging, where membrane-bound cellular structures remain unlabeled while the surrounding extracellular space is made to fluoresce to provide a negative contrast shadow image. When based on super-resolution STED microscopy, the technique in effect provides a positive image of the extracellular space geometry and entire neuropil in the field of view. Other noteworthy advantages include the near elimination of the adverse effects of photobleaching and toxicity in live imaging, exhaustive and homogeneous labeling across the preparation, and the ability to apply and adjust the label intensity on the fly. Shadow imaging is gaining popularity and has been applied on its own or combined with conventional positive labeling to visualize cells and synaptic proteins in their parenchymal context. Here, we highlight the inherent limitations of fluorescence microscopy and conventional labeling and contrast these against the pros and cons of recent shadow imaging approaches. Our aim is to describe the brief history and current trajectory of the shadow imaging technique in the neuroscience field, and to draw attention to its ease of application and versatility.

## Introduction

Seeing is believing, and in the history of science arguably no other experimental approach has been more essential than that of simple visual observation of a sample combined with analytic descriptions. It is therefore no wonder that microscopy has remained a key approach through centuries and has undergone extensive developments to refine what can be visualized by scientists across research fields. This holds particularly true for the field of neuroscience, which seeks to understand the structure and function of the billions of highly specialized and morphologically complex neural cells of the brain. Of the various microscopy techniques, fluorescence microscopy holds notable advantages for neuroscience as it reconciles multicolor capabilities, structural as well as functional imaging, and advanced labeling strategies.

Fluorophore labeling of a specimen must by design be targeted, and targeting comes with remarkable benefits, for example in terms of specificity and multicolor schemes. Yet targeting also entails major limitations and confounders because it is intrinsically inexhaustive and inhomogeneous across targets, even within a given sample.

So-called *shadow imaging* overcomes many of these limitations by offering homogeneous and exhaustive fluorescence labeling of the surroundings of cellular targets rather than the targets themselves, leaving these visible as shadows in the fluorescence. It comes with additional advantages, as well as limitations of its own, and it cannot plainly substitute targeted labeling strategies; rather, it extends what can be imaged and analyzed in fluorescence microscopy, particularly when used on high-resolution microscopes that can resolve key cell structural morphologies in live tissue, including synaptic structures in live tissue.

Below, we first outline the working principles of the most frequently applied fluorescence imaging modalities, followed by an outline of how targeted fluorophore labeling works. These sections facilitate the subsequent introduction to shadow imaging, where we describe the working principles of the approach and summarize its current applications to bioimaging.

## Fluorescence imaging modalities

The core principle of optical imaging is to discern objects of interest based on contrast in the form of differences in brightness. This contrast can be achieved through various methods, as evident from the diverse range of light-microscopy techniques (Stephens and Allan, 2003; Cox, 2012). The introduction of fluorescence microscopy revolutionized the bioimaging field by overcoming key limitations of original brightfield techniques, and in the 1940s, Coons and Kaplan (1950) made groundbreaking advancements by developing fluorescently labeled antibodies that paved the way for imaging cells and subcellular structures with molecular specificity.

Fluorescence microscopy relies on labeling samples with fluorophores that can be selectively excited and brought to emit fluorescence. One of its notable strengths is the compatibility with live cells and tissue, which enable imaging of cellular structure and dynamics across neural model systems. As a live cell far-field technique, it synergizes effectively with other methodologies, such as electrophysiology and optogenetics, facilitating scientific progress that could not be made by either modality alone. Fluorescence microscopy allows for multicolor imaging, as well as more sophisticated fluorescence lifetime imaging (FLIM) to separate fluorophores emitting at the same wavelength based on their fluorescence lifetime (Lakowicz et al., 1992), and Förster resonance energy transfer (FRET) imaging of closely situated complementary fluorophore pairs (Förster, 1965; Sekar and Periasamy, 2003).

Widefield epifluorescence microscopy is the most applied approach for imaging fixed or live cells and proteins across cell cultures, tissue slices, live animals, and various other preparations in the biosciences, in particular combined with immunochemical labeling strategies. The widefield modalities also include more advanced approaches, including super-resolution microscopy techniques such as the single molecule localization microscopy (SMLM) main incarnations Photoactivated Localization Microscopy (PALM), Stochastic Optical Reconstruction Microscopy (STORM), or variants of Point Accumulation for Imaging in Nanoscale Topography (PAINT) and saturated structured illumination microscopy (SIM). These cutting-edge methods enable imaging and single-particle tracking beyond the diffraction barrier that limits optical resolution to around 200 nm [For recent reviews (Tønnesen and Nagerl, 2013; Schermelleh et al., 2019; Lelek et al., 2021)]. In parallel, widefield illumination techniques have undergone a transformative evolution, with recent light-sheet and lattice light-sheet techniques emerging as game changers in the field (Huisken et al., 2004; Chen et al., 2014; Power and Huisken, 2017).

In the complementary laser beam-scanning approaches a collimated laser beam is focused by the microscope objective, resulting in a small focal spot called the microscope point-spread function, the size and shape of which correspond to the resolving power of the microscope. This spot is scanned across the specimen in two or three spatial dimensions and the image is assembled pixel by pixel over time. The main advantage of the beam-scanning approaches is their ability to minimize detection of out-of-focus fluorescence and crosstalk from the neighboring pixels, which indeed are core advantages of confocal and two-photon microscopy (Cremer and Cremer, 1978; Denk et al., 1990).

The advantage of good optical sectioning extends to beam-scanning-based super-resolution imaging modalities, of which stimulated emission depletion (STED) microscopy is the most widely applied (Hell and Wichmann, 1994). In essence, STED microscopy relies on confocal or two-photon microscopy to excite the fluorophores of a sample, and in addition, a laser beam with a typically doughnut shaped point-spread function is introduced. This second beam induces stimulated emission to effectively return excited fluorophores to their ground state before they spontaneously emit fluorescence, thereby depleting the excited state. As a result, only exited fluorophores in the center of the doughnut emit spontaneous fluorescence, and this localized emission can be confined to a subdiffraction volume to allow super-resolution imaging [Reviewed recently in (Lenz and Tønnesen, 2019; Calovi et al., 2021)].

## Advantages and drawbacks of targeted fluorophore labeling

Fluorophore labeling can be achieved in many ways; for example, genetic expression of fluorophores in living cells or immunohistochemical labeling in fixed tissue. Genetic fluorophore expression can be made extremely specific and selective by choosing a given gene promoter and further by directly tethering the fluorophore to a given protein of interest so that the subcellular location of this can be directly read out over space and time. Expression can further be inducible so that the fluorophore is expressed only in a specific time window. Genetic labeling has permitted radical visualization of dynamic cellular and tissue events in cell cultures and live animals and has shed light on the mechanisms of function, development, and pathology of the brain [For example (Feng et al., 2000; Madisen et al., 2010)]. Immunohistochemical labeling in fixed samples can similarly be extremely specific, and multicolor labeling can be routinely performed.

It is important to note that targeted labeling, genetic, immunochemical, or other, is essentially never exhaustive, meaning that some proteins or cells fail to be labeled even if they seemingly meet the targeting criteria (Toseland, 2013). Nonetheless, this seldom has a measurably negative impact on results as analyses usually focus exclusively on the successfully labeled targets. Conversely, many fluorescent labeling probes and vectors will not be exclusive to a single labeling target, and unintended parallel labeling of unwarranted targets may confound analyses. This may be an antibody that recognizes not only the intended protein, but to some degree also others, or it may be a promoter that is expressed to some extent in a cell type that is not the primary target of an experiment.

Differences in labeling intensities between targeted proteins or cells may reflect promoter activity in genetic fluorophore expression, or protein expression levels in immunohistochemistry. They may also reflect methodological or technical variables, such as cellular transgene expression levels, or antibody diffusional accessibility to the various targets within a sample. Quantitative analyses based on fluorescence intensity will suffer under these confounders, though experiments are usually designed taking these into account.

A tangible and impactful limitation in fluorescence imaging is fluorophore bleaching and the associated phototoxicity observed in live-cell experiments (Laissue et al., 2017). Rather than emitting spontaneous fluorescence, fluorophores in the excited state can under certain conditions, such as intense illumination and chemical interactions, undergo intersystem crossing to triplet states and their progenies. These states bring temporary or permanent bleaching by participating in photochemical reactions (Song et al., 1996). Effectively, the fluorophore molecule is destroyed and may react with proteins and cause toxicity in living cells. Bleaching and toxicity can be mitigated by minimizing excitation light illumination intensities and by including antioxidants and free radical scavengers, for example glutathione or ascorbic acid, in the perfusion solution of live-cell experiments (Icha et al., 2017).

A common strategy to identify and morphologically analyze neural cells is to genetically express freely diffusible cytosolic or membrane labels, such as green or red fluorescent protein (GFP or RFP). In this context it is worth remembering that whether observed in live cells or after fixation, genetic expression of fluorophores is always overexpression, which comes with more or less pronounced side effects from the cells having to cope with the presence of a foreign protein. Depending on the experimental paradigm, this may have negligible impact on experiment outcomes, though occasionally the effects may be more severe and may give rise to erroneous conclusions. Indeed, GFP overexpression may by itself render neural cells more vulnerable and cause labeled cells to die due to immunogenicity and toxicity of GFP (Ansari et al., 2016). Inducible vectors may to some extent alleviate such adverse effects by limiting the time period of overexpression, though it cannot eliminate them.

In certain experiments, it is desirable to genetically express a fluorophore directly fused to a protein of interest; for example, to reveal the subcellular distribution of a specific synaptic receptor subunit. However, this strategy comes with two main confounders. The first is that physical coupling of the fluorophore to the protein may impact the protein’s function; for example, its active transport or diffusional properties. For example, GFP is around 27 kDa in size (Nishiuchi et al., 1998), which is considerable compared to individual glutamatergic AMPA receptor subunits that weigh around 100 kDa (Reimers et al., 2011), or postsynaptic density scaffolding protein PSD-95 of 95 kDa, both of which are commonly tagged proteins in neuroscience studies. If antibodies rather than genetically encoded protein coupling are used to tag proteins, these will add additional size, as regular monoclonal antibodies are typically >100 kDa, and even camelid nanobodies are around 15 kDa (Beghein and Gettemans, 2017). All other things equal, fluorophore tagging can impact the effective diffusion coefficient of these corresponding to the added molecular size and thereby impact their mobility, even if this may be to some extent accounted for through control experiments.

The second major confounder is that tagged proteins are commonly overexpressed at non-physiological high levels, and many neuroscience studies analyze GFP-tagged proteins on a background of non-tagged endogenous protein expression levels. To counter this, endogenous expression can be silenced and completely replaced by expression of the fluorophore-tagged transgene under the original promoter of the protein, while still a laborious procedure, the CRISPR-Cas9 system has made such gene editing much simpler (Mali et al., 2013; Ran et al., 2013).

## Fluorescence shadow imaging with negative contrast

**FIGURE 1:**
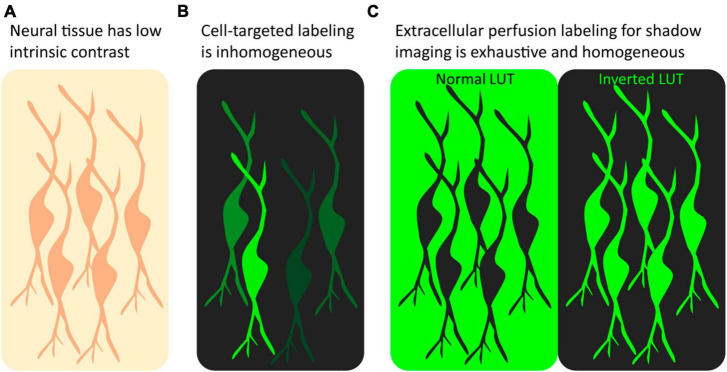
Positive label imaging vs. shadow imaging. **(A)** Unlabeled tissue has low contrast, and beyond somata few structures can be recognized. **(B)** After targeted positive labeling, e.g., by genetic fluorophore expression or immunostaining, a subset of the targeted cells will appear fluorescent at various intensities. **(C)** Perfusion with freely diffusible fluorophore labeling makes all cells in the field of view appear as shadows in the fluorescent ECS. Inverting the LUT makes the unlabeled neurons appear fluorescent at equal intensity.

The above sections have discussed targeted fluorophore labeling of samples and the associated advantages and disadvantages. However, an alternative labeling strategy, known as *shadow imaging* or imaging with negative contrast, that does not directly target the object of interest is gaining popularity in fluorescence microscopy. In this approach, the surroundings of cells rather than the cells themselves are made to fluoresce, making cellular structures visible as dark silhouettes, or shadows, on a fluorescent background. In other words, the cells of interest remain unlabeled and are made visible using negative contrast fluorescence imaging. Similar to a negative photograph, the resulting cellular shadow image contains the same information as a regular positive image, and it can be made to resemble a conventional positive contrast image by simply inverting the chosen lookup table using any basic image processing software such as ImageJ (Schneider et al., 2012; Figure 1). The strategy is not a competitor to conventional positive labeling fluorescence microscopy as it differs fundamentally in its applicability and the type of data it provides; rather it is a complementary, or even synergistic, approach that can be combined with conventional positive labeling schemes to gain additional insights and a more comprehensive understanding of cellular structures and dynamics.

The concept of shadow imaging was introduced more than a decade ago to facilitate *in vivo* patch-clamp electrophysiology experiments by injecting a fluorophore from the patch pipette during its navigation toward a neuron during two-photon microscopy (Kitamura et al., 2008). The cell bodies in the vicinity of the pipette, from which fluorescence solution would spill out, would emerge as apparent shadows that could be targeted for patch-clamp recordings, with the added advantage that after patching the fluorophore would effectively label the cell. The approach allowed identification of putative cortical pyramidal neurons and interneurons based on coarse morphological characteristics such as soma size and presence of apical dendrites. However, due to the diffraction-limited optical resolution of the two-photon microscope, the technique provided limited morphological information beyond these larger cellular structures. As a result, the shadow technique in fluorescence microscopy has been used primarily by a small group of researchers as a means for patch-clamping neurons rather than for structural imaging [e.g., (Kitamura et al., 2008; Noguchi et al., 2011)]. Ten years after the original shadow-patching publication, we took part in the next notable step toward establishing the fluorescence shadow imaging approach as a method for imaging cellular structure in tissue and cell cultures. Our starting point was our previous work with STED microscopy that can achieve nearly 1,000-fold better volume resolution than two-photon microscopy and which we used to study positively labeled dendritic spines (Tønnesen et al., 2011a,^2014^). Adopting the shadow imaging approach, the improved resolution of STED microscopy allowed us to largely resolve the geometric structure of the neuropil in organotypic brain slices after perfusion labeling with a hydrophilic fluorophore that distributes homogeneously by diffusion in the interstitial space. The result is a super-resolved image of the neuropil at synaptic scale resolution and inherently a corresponding positive image of the extracellular space (ECS) structure (Tønnesen et al., 2018). We dubbed this approach super-resolution shadow imaging (SUSHI), and while it was spurred by the very high optical resolution of our 3D-STED microscope, recent efforts point to the method being more generally applicable across microscopy modalities, and becoming increasingly versatile.

## Main differences between shadow and positive label fluorescence imaging

The shadow imaging approach is a straightforward microscopy approach that relies on the classic principles of fluorescence microscopy, though it operates in a negative contrast regime whereby the observed cellular structures remain unlabeled.

Several defining traits differentiate it from direct positive labeling schemes. Perhaps the main difference is that shadow imaging based on perfusion labeling with freely diffusible fluorophores yields an all-inclusive image of the membrane-bound cellular structures in the field of view. This is in stark contrast to cell-targeted labeling where cells or proteins within a sample are labeled to various extents, sometimes leading to the identification of false-positive and false-negative targets. The homogeneous all-inclusive label of shadow imaging, combined with the spatial continuity of the ECS across its extent, comes with two important advantages.

Firstly, the confounding issue of incomplete labeling associated with positive labeling strategies is eliminated. In the shadow image, if a membrane-bound structure is not visible in the image, it is because it genuinely does not exist, not simply because it is unlabeled.

Secondly, a diffusible fluorophore added to the bath perfusion distributes homogeneously in the entire interstitial fluid and ECS, and differences in fluorescence intensity in an image directly reflect the ratio of cellular (non-fluorescent) to extracellular (fluorescent) structure in each pixel. This is again in contrast to targeted positive labeling methods, for example, genetic or antibody based, where individual cells commonly differ greatly in their fluorescence intensity, reflecting a mix of biological and experimental variables that are commonly impossible to untangle. An example of the stochastic fluorophore expression levels resulting from genetic fluorophore labeling is the Brainbow labeling strategy that exploits this variation to the fullest (Livet et al., 2007). Accordingly, and in contrast to positive labeling approaches such as Brainbow, shadow images allow quantitative analyses of intensity levels, for example, to quantify the volume of the ECS in a given image frame, a strategy that can be used to effectively map out the ECS volume fraction at the spatial resolution offered by the applied microscope (Tønnesen et al., 2018; Dembitskaya et al., 2023).

## Shadow imaging across microscopy modalities

We have mentioned two-photon-microscopy-based shadow imaging and in particular SUSHI, though the negative contrast shadow imaging approach is indeed generally applicable and is gaining traction outside the field of super-resolution and STED microscopy.

The shadow imaging approach started as a two-photon microscopy approach for patch-clamp experiments, and the fluorescence imaging community now seems to have come full circle, as two-photon shadow imaging was recently published as an interesting *in vivo* imaging modality in its own right (Dembitskaya et al., 2023). The original SUSHI paper already describes two-photon shadow imaging in organotypic slices, revealing structural details of remarkably thin cellular processes to provide neuropil images. Beyond neuroscience, two-photon shadow imaging has been used to image bone marrow and lymph nodes, highlighting its applicability for intravital imaging (Wu et al., 2021). It therefore appears that the two-photon based approach could be generally adopted by existing two-photon microscopy users and be highly applicable to *in vivo* and *ex vivo* shadow imaging experiments.

The recent paper by Dembitskaya et al. (2023) further presents images obtained using confocal microscopy-based shadow imaging in organotypic slices, where dynamics of positively labeled GFP-expressing microglia are analyzed with reference to the surrounding neuropil visualized by shadow imaging. It is notable how much ECS structural detail can still be visualized by diffraction-limited confocal microcopy in the presented images, where the difference between SUSHI and confocal shadow images is difficult to grasp with the naked eye. The same paper proceeds to introduce light-sheet microscopy-based shadow imaging in organotypic brain slices in combination with deconvolution and image artifact cleaning steps as a method of acquiring higher frequency frame rates than beam-scanning approaches allow. The tradeoff for the speed of the light-sheet approach is a more complex dual microscope objective setup and slightly lower contrast images than confocal microscopy-based shadow imaging, though the light-sheet images still reveal a wealth of neuropil and ECS structure. Another recent report combined SUSHI of neurons with confocal microscopy of genetically expressed synaptic proteins, including synaptophysin-1 and PSD-95 (Velicky et al., 2023).

These successful combinations of different imaging modalities showcase the cross-modality versatility of shadow imaging, enabling comprehensive analysis of neuronal morphologies and synapses in conjunction with other imaging techniques.

A related complementary way of visualizing the ECS structure, and indeed the first super-resolution based approach applied to the ECS, is by tracking single-walled carbon nanotubes diffusing in the ECS (Godin et al., 2017). If these diffuse over sufficient time to explore the complete ECS of interest, their trajectories effectively provide an ECS structural image and thereby a shadow image of the neuropil. While the technique does not offer the same homogeneous labeling intensity across the entire field of view as when using smaller freely diffusible fluorophores, it is an interesting technique because it facilitates parallel structure and diffusion analyses, and it could be interesting to combine it with SUSHI in future experiments.

## Shadow imaging in live preparations

The original shadow imaging approach is based on the diffusional spread of fluorophore in the ECS, and this aspect of the technique comes with several benefits for experimenters. It allows continuous replenishment of bleached fluorophores to practically eliminate the effects of bleaching in the resulting images. Photo-toxicity is correspondingly much lower than in positive labeling schemes, as any generated reactive molecular species diffuse away from the imaged area and dilute, instead of being trapped inside living cells where they may cause acute toxicity. Indeed, the perfusion labeling approach allows experimenters to vary laser powers as well as fluorophore concentrations as a means to increase or decrease signal intensity, facilitating the use of very low laser power. This may prove interesting for quantifying if it is advantageous to have higher fluorophore concentration and lower excitation light intensity, or *vice versa*, in the context of photo-toxicity.

**FIGURE 2:**
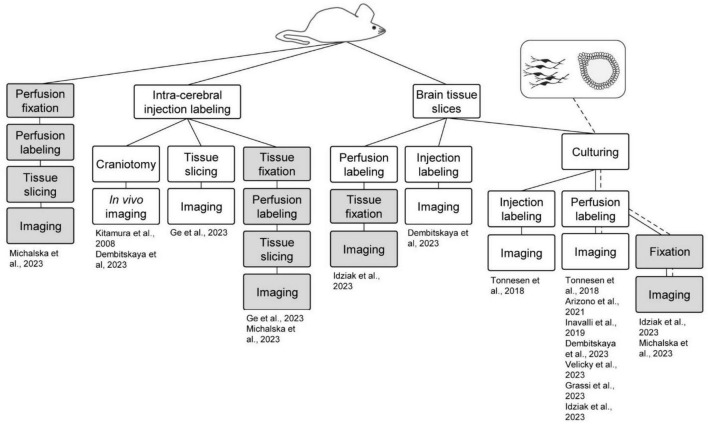
Shadow imaging in different model systems. Shadow imaging for neuroscience has been performed across sample preparations, spanning *in vivo* labeling and imaging, brain slices, cell cultures, brain organoids, and fixed sample preparations. Green boxes represent live tissue/culture steps, while red ones denote fixed samples. The work flows are clustered based on the starting preparation being an animal or cell/organoid culture.

Another advantage of ECS perfusion labeling is that the fluorophore label is homogeneously distributed at equimolar concentrations in the perfusion solution and throughout the interstitial fluid, which sets the basis for quantitative analyses, as described above. Perfusion labeling can furthermore be performed simply by adding a fluorophore to the perfusion at any point of an experiment, and the concentration of the fluorophore in the perfusion solution can be adjusted on the fly during experiments to optimize it for various factors such as imaging depth, laser intensities, detector sensitivity, intensity of complementary second fluorophore, and so forth. Similarly, at the end of the experiment, the fluorophore can be washed out in a matter of minutes to leave negligible background (Tønnesen et al., 2018; Dembitskaya et al., 2023). In the context of SUSHI, all existing live sample preparations can conceivably be adopted, and the need for time-consuming targeted labeling steps that are incompatible with acute labeling can be eliminated. In the following sections we highlight the preparations that have been imaged using the shadow approach and further summarize these in Figure 2.

## *In vivo* SUSHI in live animals

**FIGURE 3:**
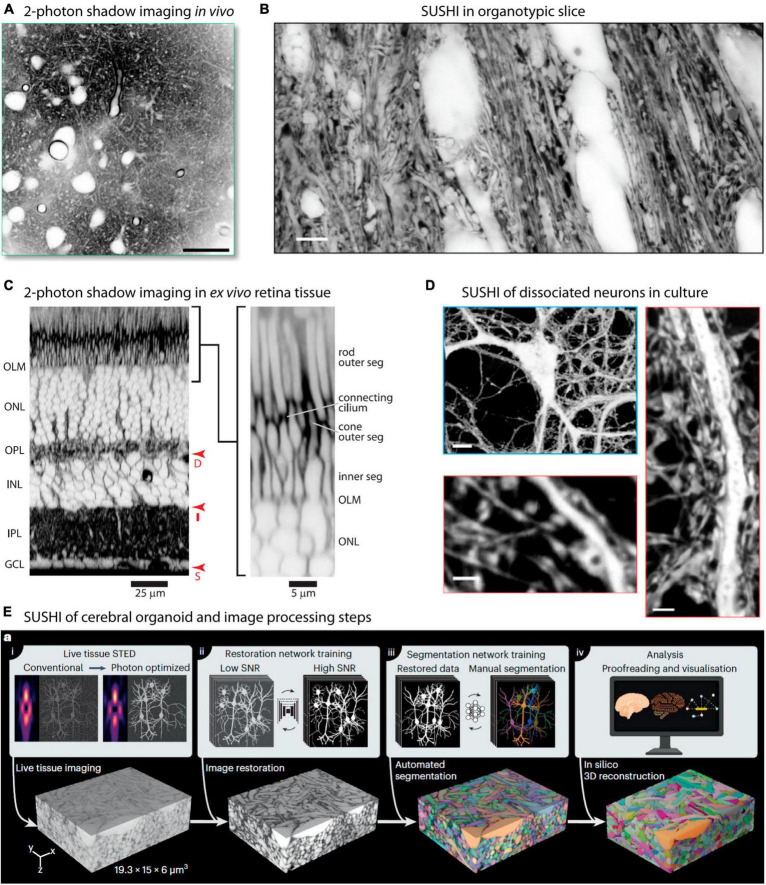
Super-resolution and two-photon shadow imaging in different live tissue preparations. **(A)** Two-photon shadow imaging of cortical neuropil of live mouse imaged *in vivo*. Scale bar is 25 μm. From Dembitskaya et al. (2023), with permission. **(B)** Raw SUSHI image of neuropil in live organotypic mouse hippocampal slice. Scale bar is 5 μm. From Tønnesen et al. (2018), with permission. **(C)** Acute mouse retina preparation imaged by two-photon shadow imaging, with the retinal cell layers clearly distinguishable. From Kuo et al. (2020), with permission. **(D)** Live SUSHI of unlabeled primary dissociated mouse brain neurons, with zoomed in views of dendritic stretches with pre- and postsynaptic structures visible. Scale bars are: top 10 μm, bottom left 4 μm, bottom right 2 μm. From Tønnesen et al. (2018), with permission. **(E)** Live human cerebral organoid imaged by SUSHI, including the off-line work flow used for image processing and tissue reconstruction. From Velicky et al. (2023), with permission.

As mentioned, the original implementation of shadow imaging was in *in vivo* settings, with the aim of targeting and patch-clamping neurons. Two-photon shadow imaging was more recently applied *in vivo* to image the cortical neuropil in living anesthetized mice after injection of Alexa Fluor 488 into the lateral ventricle from where it disperses to other parts of the brain (Dembitskaya et al., 2023; Figure 3A). While the two-photon based approach does not offer as high spatial resolution as the SUSHI approach, it still readily renders somata and dendrites visible, allowing extraction of morphological information, as was also evident from the original two-photon shadow images from Kitamura et al. (2008). *In vivo* shadow imaging holds great promise for contributing to delineating how diffusion and bulk flow may contribute to brain metabolite clearance and understanding whether the ECS dynamics observed in brain slices can indeed be found in the intact brain.

## Organotypic brain slices

Organotypic brain slices are particularly attractive for SUSHI because beyond 1 week in culture any damaged cells will have either died and disappeared or healed, such that any cells remaining in the slice are intact and no slice surface damage remains. At the same time, the tissue layering, neural process organization, synaptic connectivity, and intrinsic neuronal properties are largely conserved, offering an opportunity to study these in a highly reproducible and accessible preparation (Figure 3B; De Simoni et al., 2003; Tønnesen et al., 2011b). Slices further allow imaging of structures that are too deep below the brain surface to reach using two-photon microscopy *in vivo*.

Organotypic slices cultured on glass coverslips according to the Gähwiler (1988) roller-drum method are excellent for inverted microscopes since the slices can be imaged directly through the coverslip. The alternative organotypic preparation, the membrane interface culture by the Muller technique, is harder to transfer and position in an inverted microscope imaging chamber due to the soft membrane they grow on, and they lack the optical benefits of cultures grown on a glass interface (Stoppini et al., 1991; De Simoni and Yu, 2006). However, they are in our experience easier to maintain in good health than Gähwiler style ones, and they have been imaged on inverted microscopes by SUSHI by placing the slice on the membrane upside down in an imaging chamber to investigate ECS structure across the hippocampal layers (Dembitskaya et al., 2023; Grassi et al., 2023).

A disadvantage of organotypic brain slice cultures is that they are by necessity prepared from 5 to 10-day old rodent pups, so there are limits to the diseases and conditions that can be modeled experimentally. Further, cultured organotypic slices undergo more or less subtle rearrangements of the neuropil as cellular processes are cut, and microglia tend to go into an activated state, even if this can to some extent be recovered (Czapiga and Colton, 1999).

## Acute tissue preparations

The acute, or *ex vivo*, slice preparation is a widely used preparation in neuroscience research, and allows researchers to investigate the complex neural networks of the brain while providing an accessible and controlled environment for various physiological, pharmacological, and imaging studies. One of the challenges of using acute brain slice for imaging, particularly in shadow imaging, is the surface damage that occurs during the slicing process. Acute brain slices are typically cut to a thickness of 200–400 μm, and this cutting inevitably damages neural cells near the cutting surface. Due to this damage, it is necessary to image at greater than around 50 μm tissue depth, where there is less damage and the neuropil remain more intact.

Nevertheless, neurons commonly branch extensively and may extend their processes hundreds of microns in all directions. Neural processes from somata located deep in the slice might therefore still branch to the cutting surface, resulting in the presence of damaged neurons throughout the acute slice preparation, even if these are fewer with increasing depth. In our experience, the cutting damage is manifested as a gradient through the slice, with the steepness of the gradient depending on the quality of the experimental procedure and the angle of the cut with respect to the orientation of the resident neural cells.

A recent paper applied two-photon shadow imaging to visualize the mouse retina in an acute *ex vivo* tissue preparation, revealing the different cell layers and their response to an osmotic challenge at high resolution (Kuo et al., 2020; Figure 3C). The retina provides a unique preparation as it is inherently accessible to visible light and does not require slicing, but can be imaged in near intact form after isolation by dissection. Two-photon shadow imaging has additionally been used to visualize the ECS in different acute mouse brain slice preparations, including hippocampus, corpus callosum, and cerebellum, which remain challenging to reach by *in vivo* imaging approaches (Dembitskaya et al., 2023).

The achievable optical resolution of two-photon shadow imaging remains diffraction limited. However, when using high numerical objectives in acute slices it enables information-rich images with structural details that are more difficult to observe *in vivo*, and the acute slice is therefore an attractive preparation for bridging the gap between organotypic slice cultures and *in vivo* experiments.

## Dissociated cell cultures

Dissociated cultured neurons provide a simplified and accessible model system for studying fundamental aspects of neural function, making them an invaluable tool in neuroscience research. The controlled environment, reproducibility, and ease of handling make them particularly well-suited for various imaging methods. Cells can conveniently be grown directly on glass coverslips to facilitate imaging using various microscopy techniques, including SUSHI. SUSHI of live culture preparations is straightforward and enables morphological analysis of pre- and postsynaptic neurons without the necessity of labeling them (Tønnesen et al., 2018; Inavalli et al., 2019; Figure 3D).

## Cerebral organoids

Organoids are three-dimensional lab-grown tissue structures based on 3D cell culture methods combined with added extracellular matrix and other molecular cues, and which recapitulate some aspects of brain tissue development. Organoids offer exciting opportunities for delving into human organ development, and brain organoids hold great promise for investigating the complexities of human brain development in health and disease (Kim et al., 2020; Eichmüller and Knoblich, 2022). SUSHI has been applied to reconstruct the volume of a living human brain organoid grown *in vitro* (Figure 3E), demonstrating the potential of this technique (Velicky et al., 2023).

## Multicolor shadow imaging in live tissue

A defining feature of fluorescence microscopy is the possibility of obtaining dual- or multicolor images by imaging different fluorophores in parallel. Shadow imaging is readily compatible with multicolor approaches, with the best color discrimination obtained when the different fluorophore species are labeling different targets.

**FIGURE 4:**
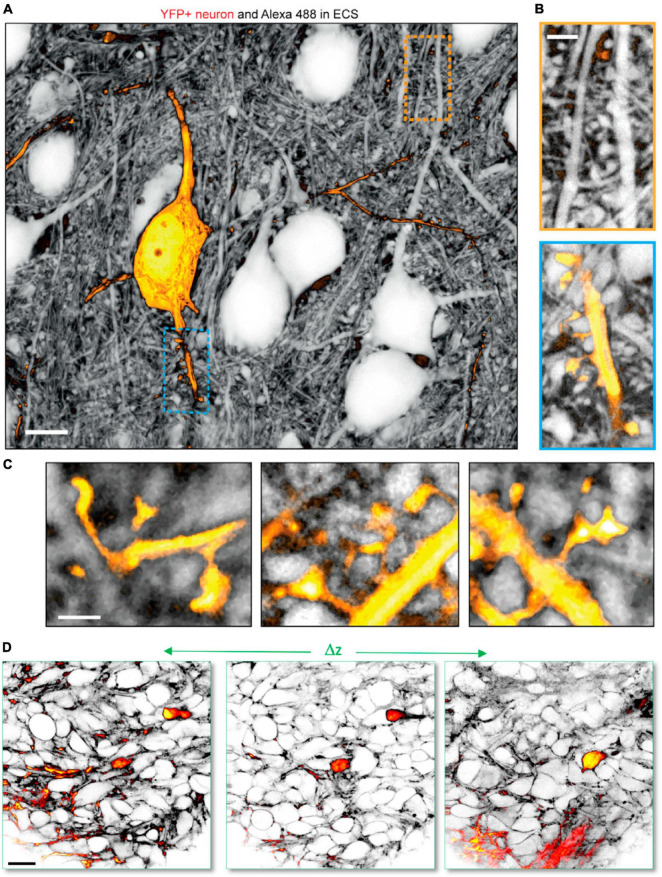
Two-color shadow imaging. **(A)** YFP labeled pyramidal neuron (yellow) imaged in the neuropil context by SUSHI based on two-color 3D-STED microscopy in live organotypic slice. Scale bar is 10 μm. **(B)** Zoom-ins on (top) unlabeled dendritic segment with presumed spines and synapses. (Bottom) YFP labeled dendritic segment with dendritic spines in the context of the surrounding neuropil. Scale bar is 2 μm. **(C)** Further zoom-in on YFP labeled dendritic spines forming synapses with unlabeled axonal boutons imaged by SUSHI. Scale bar is 1 μm. **(A–C)** From Tønnesen et al. (2018), with permission. **(D)** *In vivo* two-color two-photon shadow imaging of GFP-expressing tumor cells injected into the mouse brain cortex, where they can be observed among unlabeled endogenous neural cells. From Dembitskaya et al. (2023), with permission.

For example, combining ECS perfusion-labeling-based shadow imaging with positive labeling of cells has been successfully performed to analyze the ECS around individual neurons (Tønnesen et al., 2018; Dembitskaya et al., 2023), astrocytes (Tønnesen et al., 2018; Arizono et al., 2021), and microglia (Dembitskaya et al., 2023) using two-color STED microscopy with spectrally overlapping fluorophore pairs, such as YFP and calcein in live tissue. This approach allows analysis of individual cells within their parenchymal context, and we previously used this to stoichiometrically analyze unlabeled presynaptic boutons with respect to postsynaptic dendritic spines on positively YFP-labeled neurons (Tønnesen et al., 2018), which would not be feasible using positive labeling (Figures 4A–C). Further, as mentioned above, SUSHI and confocal shadow imaging have been combined in a sample with genetic expression of synaptic markers to allow two- or three-color combined STED and confocal imaging to reveal the neuropil context around the synaptic markers (Velicky et al., 2023). Two-color shadow imaging was recently used to visualize GFP-labeled tumor cells and unlabeled cortical neurons by *in vivo* two-photon microscopy (Dembitskaya et al., 2023), providing proof of concept for visualizing the invasion and displacement of healthy parenchyma by cancerous cells (Figure 4D).

Two freely diffusible fluorophore species can be simultaneously added to the ECS by perfusion, and their respective distributions analyzed; however, as the species mix in the perfusion solution it is not straightforward to separate their emission spectra if they overlap.

We previously used two spectrally overlapping fluorophore species to investigate whether differently sized fluorophores would distribute differently in the ECS, for example as a result of sieving by the ECS geometry or the extracellular matrix, though this appeared preliminarily not to be the case (Tønnesen et al., 2018). Such experiments with mixed fluorophores could benefit from using spectrally separate fluorophores, though this necessitates a designated excitation laser for each, which complicates the STED setup and requires more photons to generate a single two-color image than if they are imaged using a common excitation and depletion beam pair (Tønnesen et al., 2011a). Conceivably, fluorescence lifetime imaging (FLIM) could allow discrimination of spectrally overlapping fluorophores based on their lifetime, but here too, mixing of the fluorophore populations may confound analyses. FRET would be another strategy that could be useful here.

The use of a freely diffusible homogeneously distributed ECS fluorophore label comes with a particular advantage when imaging the ECS and positively labeled cells in parallel. Individual positively labeled cells and the ECS can readily be imaged in the same color by a single detector, and the resulting image can then be processed into a two-channel image based on fluorescence intensity discrimination. This is possible, and practically trivial, because positively labeled cells (e.g., GFP or YFP expressing neurons) often appear very bright, and the fluorescence intensity of the perfusion ECS label can be easily reduced so that the ECS fluoresces at much lower intensity to allow simple separation of the structures based on brightness. In the image brightness/contrast settings of image processing software, it is simple to adjust the pixel intensity histogram to eliminate the weaker ECS and see only the labeled cell. This can then be merged with an inverted duplication of the same image including the full histogram to effectively yield a dual channel image. The higher the spatial resolution of the scanning microscope, the better the technique works, though in our hands the approach also works very well for regular two-photon microscopy on a commercial setup. Many positively labeled cells can be imaged in parallel on an ECS background if the brightness of the cells is higher than that of the ECS label. In principle, the approach can also be extended to obtain images with three (or more) channels using dual-color imaging on two detectors plus intensity discrimination for the third channel.

## Combining shadow imaging with functional optical approaches

Fluorescence microscopy is routinely used not for imaging structure, but for reading out functional dynamic signals and perturbing neural function. The spectral specificity of fluorophores and of functional optical probes permit their co-application in a single experiment, and structural and functional fluorescence microscopy are often combined.

**FIGURE 5:**
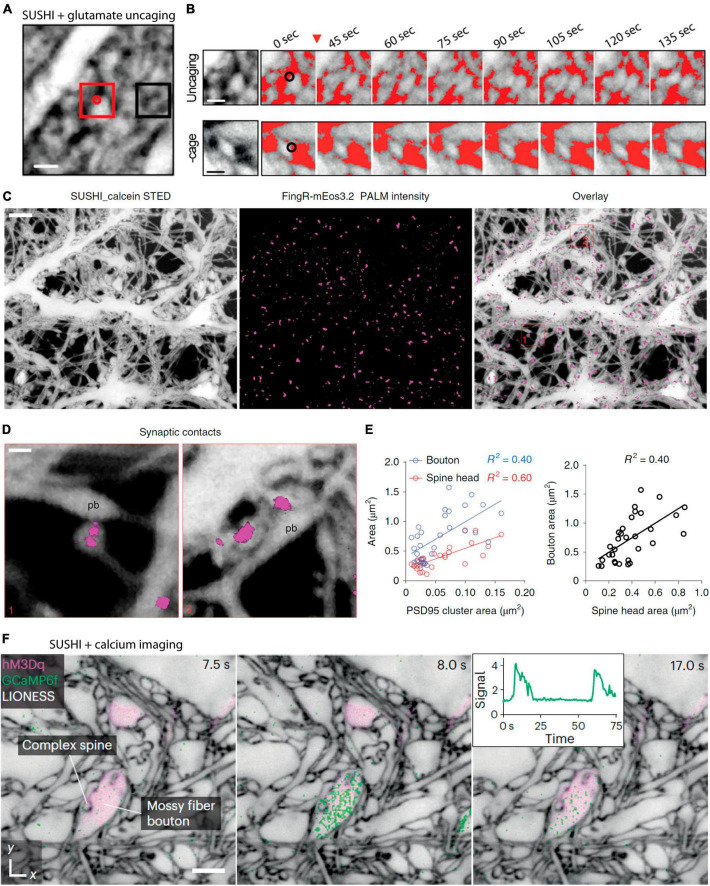
Combining shadow imaging of cellular structure with functional optical techniques. **(A)** Shadow imaging of hippocampal slice culture neuropil while locally two-photon uncaging glutamate. Glutamate is uncaged at the red dot. **(B)** Zoomed-in time lapse of the uncaging area of **(A)** showing local remodeling of the neuropil that is not observed under control conditions without the caged glutamate. Scale bars are 1 μm. From Tønnesen et al. (2018), with permission. **(C)** SUSHI of primary dissociated neurons combined with single particle tracking PALM of endogenous PSD-95 (Magenta) labeled by FingR_mEos3.2, and the merge of the PSD-95 protein onto the SUSHI morphological image Scale bar is 2 μm. **(D)** Zoom-ins showing postsynaptic PSD-95 on super-resolved dendritic spines imaged by SUSHI. **(E)** The combination of these two super-resolution approaches enable geometric comparisons of spine structure with PSD-95 size, as well as spine and bouton size comparisons. The shadow approach greatly facilitates these experiments as it circumvents the need to positively label the pre and postsynaptic neurons. Scale bar is 1 μm. From Inavalli et al. (2019), with permission. **(F)** Calcium imaging using GCaMP6F following pharmacological activation of specific neurons by DREADDs via excitatory hM3Dq receptors. The three frame time-lapse show mossy fiber bouton calcium dynamics on the backdrop of a static frame neuropil imaged by SUSHI. The insert depict the calcium transient over time. Scale bar is 2 μm. From Velicky et al. (2023), with permission.

Our previous work took advantage of two-photon mediated glutamate photolysis, or photo-*uncaging*, to locally release glutamate in the neuropil and image the dynamic response by SUSHI. We found that glutamate uncaging at levels mimicking synaptic activity would induce local remodeling of the neuropil at the release site, though it remains unknown which cells are involved in this response (Tønnesen et al., 2018; Figures 5A, B).

We were also previously involved in combining SUSHI with super-resolved single-particle tracking PALM and uPAINT for multimodal multicolor imaging to reveal the trajectories of PSD-95 proteins and glutamatergic receptor subtypes diffusing on dendritic spines visualized by SUSHI (Inavalli et al., 2019; Figures 5C–E). This approach benefited from the SUSHI approach being applicable to dissociated cell cultures and combining scanning and widefield super-resolution approaches in a single imaging platform.

Super-resolution shadow imaging has further been used consecutively with conventional confocal imaging of intracellular calcium transients as reported by the GCaMP6f genetically encoded calcium indicator (Velicky et al., 2023; Figure 5F) and a newly introduced low-affinity genetically encoded calcium indicator, GreenT, that localize to the cell membrane and reportedly respond to changes in interstitial calcium (Valiente-Gabioud et al., 2023).

In essence, shadow imaging is a versatile approach across fluorescence microscopy modalities that enables unbiased all-inclusive images of cellular structures in the field of view. It allows imaging of structural dynamics in live tissue and can be combined with immunohistochemistry and volumetric tissue-expansion procedures in fixed tissue, as well as functional fluorescence microscopy approaches in live tissue.

## Shadow imaging in fixed tissue preparations

**FIGURE 6:**
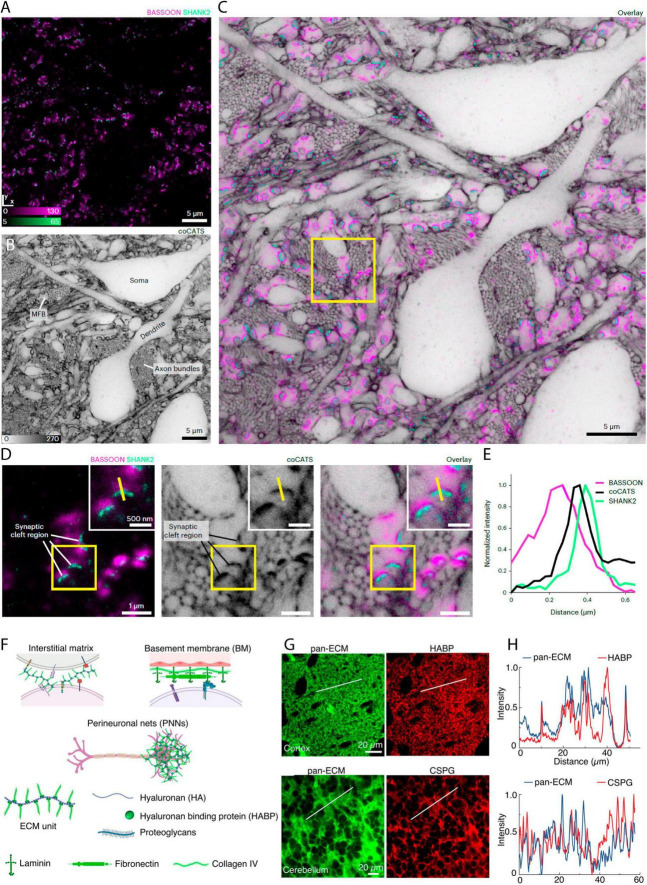
Shadow imaging in fixed tissue preparations. **(A)** Immunostaining for presynaptic label Bassoon (confocal, magenta) and postsynaptic protein Shank2 (STED, green) in fixed brain tissue, **(B)** with the corresponding frame shadow imaged by STED after injection of a NHS-coupled diffusible fluorophore into the lateral ventricle *in vivo* (Here labelled coCATS). **(C)** The merged image show the synaptic proteins along with the neuropil context. **(D)** Zoom-ins of a single frame showing the immunostaining or shadow image of the neuropil, as well as the merged image. **(E)** Line intensity profile of the yellow line in **(B)** that provide distance information of the given pre-/post-synapse pair. From Michalska et al. (2023), with permission. **(F)** ECS labeling with NHS-coupled fluorophore binding to extracellular matrix protein. The brain extracellular matrix is heterogeneously distributed across and within brain regions and is present in different compositions and forms in the interstitial matrix, vascular basement membrane (BM), and perineuronal nets (PNNs). Its primary constituents are hyaluronan, hyaluronan binding protein (HABP), proteoglycans, laminin, fibronectin, and collagen. **(G)** NHS coupled Atto-488 injected *in vivo* show good agreement in distribution with HABP, as well as with chondroitin sulfate proteoglycans (CSPG), as evident from the displayed fixed tissue immunostainings, **(H)** and the line profiles of the merged Atto-488 and immunostainings. A notable confounder is that both immunotargets, and the NHS binding sites, are highly prominent throughout the ECS, and therefore large overlaps in fluorophore/target distributions are expected, even if the Atto dye does not specifically label matrix proteins. From Ge et al. (2023), with permission.

Alongside the advantages of shadow imaging in live tissue, there are great advantages to being able to work with fixed tissue, and SUSHI has recently been adapted to this setting. Among the major advantages, as an alternative to super-resolution microscopy, subdiffraction structures can be brought into the reach of diffraction-limited microscopy modalities by chemically expanding the fixed sample volumetrically before imaging, as has been recently reported (Michalska et al., 2023). Labeling the ECS using a biotinylated [poly-ethylene-glycol-12]-[NHS] molecule allowed visualization of cellular structures by confocal microscopy after fourfold volume expansion of the organotypic hippocampal slices and streptavidin-coupled fluorophore labeling of biotin (Michalska et al., 2023). The same publication introduces general strategies for SUSHI in fixed brain tissue, thereby enabling its combination with immunohistochemistry to allow mapping of proteins of interest, for example synaptic proteins Bassoon and SHANK2, onto cellular morphologies imaged by SUSHI (Michalska et al., 2023; Figures 6A–E). The basic principle of the reported fixed tissue approach is labeling of the extracellular space by introducing dyes coupled to small hydrophilic molecules, for example, fluorophore coupled *N*-hydroxysuccinimide (NHS) esters that covalently bind to primary amines in the ECS. As the targeted amines are ubiquitously found throughout the ECS, the resulting distribution is reportedly homogeneous enough to produce SUSHI images of the neuropil.

Interestingly, this assumption of homogeneity has been recently put into perspective. The authors of a recent paper adopt a similar approach of injecting an NHS-coupled fluorophore in the live mouse brain and later image this by STED microscopy after tissue slicing. Yet, in this latter study, the authors present the approach as a way of imaging specifically the extracellular matrix, rather than the ECS (Ge et al., 2023; Figures 6F–H). As the matrix is indeed not homogeneously distributed in the ECS (Dauth et al., 2016), it remains to be determined whether ECS analyses based on amine-binding fluorophore reporters will confound analyses of ECS geometry compared to corresponding images based on freely diffusible fluorophores. If indeed NHS and other coupled fluorophore reporters with affinity for extracellular molecules can to some extent reveal the extracellular matrix distribution, such labeling offers an interesting approach to combine with a freely diffusible ECS label, with the potential to delineate the distribution of the extracellular matrix within the optically resolved ECS.

In the context of tissue fixation, it is interesting that another report found that chemical PFA-based fixation of organotypic slice cultures has a minimal impact on ECS volume and structure, with the only discernible effect being a modest impact on dendritic spine structure (Idziak et al., 2023). This is in contrast to more extended fixation protocols that additionally incorporate slicing and tissue mounting, which reportedly had pronounced effects on dendritic spine neck geometry as well as on ECS volume and structure in electron microscopy images (Korogod et al., 2015; Tamada et al., 2020).

## Analysis considerations

Shadow images based on perfusion or injection of a freely diffusible hydrophilic fluorophore reveal both the structure of the ECS and that of the resident cells in a *yin-yang* manner. The ECS is labeled and imaged directly and can be analyzed by conventional means, whereas the cellular compartment appears as shadows, such that investigators cannot simply use the same analysis steps as for analyzing positively labeled structures. Fortunately, this obstacle is easy to overcome, with the simplest way a mere inversion of the shadow image lookup table prior to analysis, with special attention paid to preserving the full pixel-intensity histogram. An established way of measuring fluorescently labeled structures that are resolved but near the resolution limit of the microscope is to extract the full width at half maximum (FWHM) of a Gaussian fit to the line intensity profile through the structure (Cole et al., 2011). Variations of the Gaussian fit approach are common, with small but notable differences between them (Lenz and Tønnesen, 2019). The FWHM analysis allows widths of individual ECS channels to be measured, as well as widths of unlabeled cellular shadows by inverting these to effectively fit an upside-down Gaussian plot to the line intensity profile. As a measure of ECS dynamics that do not directionally increase or decrease ECS volume over time, we previously performed a pixel-by-pixel standard deviation analysis across time lapses, which identified where in the timelapse frames ECS remodeling occurred and to what extent (Tønnesen et al., 2018).

The extracellular space volume fraction can be analyzed via image thresholding and binarization procedures, of which several have been proposed (Tønnesen et al., 2018; Dembitskaya et al., 2023), or by a more analog integration of the fluorescence across the image plane without binarization (Tønnesen et al., 2018). The various approaches yield volume fractions in the range predicted by electron microscopy and electrodiffusion studies [as we recently reviewed in (Soria et al., 2020)], though differences remain in these measurements that warrant further investigation.

Synaptic scale reconstructions of unlabeled cells in SUSHI images have been created manually based on thresholding and surface rendering in ImageJ (Tønnesen et al., 2018), and more recently through an extensively automated workflow that allows reconstruction of the full imaged tissue volume to around 80% agreement with ground truth when analyzing dendritic spines (Velicky et al., 2023). In the latter paper, Velicky et al. present a complete pipeline for the facilitated image processing and tissue reconstruction of neural morphologies in SUSHI images, including identification of synapses based on co-registering SUSHI images with confocal images of synaptic proteins as mentioned above. The workflow takes advantage of tissue reconstruction approaches developed for electron microscopy, which has undergone extensive developments toward large-scale tissue reconstructions.

## Outlook for fluorescence microscopy shadow imaging

The shadow imaging approach is steadily gaining momentum in neuroscience because of its versatility and ability to visualize structures that are out of reach of targeted fluorophore labels. After being somewhat overlooked as a structural imaging approach since its introduction as a two-photon microscopy-based modality to guide patch-clamp experiments in Kitamura et al. (2008), the advent of SUSHI again brought it into focus in Tønnesen et al. (2018), and since then several preprints and peer-reviewed articles have emerged spanning various fluorescence microscopy modalities, sample preparations, and experimental paradigms. Most of these are still focused on method development, and we accordingly foresee more applied use of the shadow imaging strategy to usher in new biological insights on the near horizon.

The potential to bring new insights in live or fixed tissue is significant, both as a standalone technique and in conjunction with complementary optical and other approaches. This is because shadow imaging uniquely provides an all-inclusive unbiased picture of the field of view, thereby opening the door to surprising discoveries that may be difficult to hypothesize and experimentally pursue based on conventional cell or protein targeted fluorescence labeling. When protocols for fixed tissue labeling become more established, shadow imaging could become a mainstay approach combined with any immunostaining, serving as a superior counterstaining to HOECHST or DAPI nuclear labels currently used to assess cell numbers. Functional imaging of cellular and extracellular calcium, membrane voltages, and other gradients will be interesting in the context of both pre- and postsynaptic partners, not to mention multipartite synapses that can be more completely visualized as the respective partners need not all be positively labeled.

Another interesting application is diffusion modeling through ECS structure, which is currently based exclusively on fixed tissue ECS reconstructions from electron micrographs, with the confounders that follow, or on representative synthetic ECS lattice structures that largely fail to incorporate the expected enormous variability of the ECS geometry at the nano- to microscale, where signaling events are likely most interesting and relevant to understand (Soria et al., 2020). Now, such modeling can be based on nanoscale resolution images of live ECS and neuropil structures, with possibilities for experimental validation of the models by imaging diffusion in live tissue. In relation to this, and to electron microscopy in general, it will be interesting to directly compare SUSHI images of the neuropil to electron micrographs, which is now possible. Furthermore, correlative light and electron microscopy (CLEM) will potentially benefit from the shadow imaging approach as the neuropil that is normally imaged by the electron microscopy part of the CLEM approach can now also be assessed by the light-microscopy modality to corroborate and synergize with the results brought about by the combined approach (de Boer et al., 2015). These and many other applications of shadow imaging using inverted contrast in fluorescence microscopy are likely to contribute to key discoveries about the structure and function of the brain in health and disease, particularly aspects relating to the ECS compartment that have been difficult to experimentally address in live tissue.

As a final remark, trying shadow imaging on a regular confocal or two-photon scanning microscope is as simple as it appears, and is something anyone working in live tissue or cell culture can do with negligible investment of time and resources. Shadow imaging can be performed by simply adding around 10–100 μM of calcein or other common off-the-shelf hydrophilic fluorophore to the perfusion solution or directly in the imaging chamber, and it is therefore something that we encourage experimentalists to try out during their normal experiments.

## Author contributions

VI: Conceptualization, Visualization, Writing – original draft, Writing – review and editing. VP: Writing – review and editing. JD: Writing – review and editing. JT: Conceptualization, Funding acquisition, Supervision, Visualization, Writing – original draft, Writing – review and editing.
